# Antiretroviral choice and severe disease predict poorer neuropsychological outcomes in HIV+ children from Africa

**DOI:** 10.3389/fped.2022.899002

**Published:** 2022-08-03

**Authors:** Lee Fairlie, Miriam Chernoff, Mark F. Cotton, Mutsa Bwakura-Dangarembizi, Avy Violari, Itziar Familiar-Lopez, Linda Barlow-Mosha, Portia Kamthunzi, Katie McCarthy, Patrick Jean-Philippe, Barbara Laughton, Paul E. Palumbo, Michael J. Boivin

**Affiliations:** ^1^Wits Reproductive Health and HIV Institute, Faculty of Health Sciences, University of the Witwatersrand, Johannesburg, South Africa; ^2^Center for Biostatistics in AIDS Research, Harvard T.H. Chan School of Public Health, Boston, MA, United States; ^3^Family Centre for Research with Ubuntu, Department of Paediatrics and Child Health, Tygerberg Hospital, Stellenbosch University, Tygerberg, South Africa; ^4^Harare Family Care CRS, College of Health Sciences Clinical Trials Unit, University of Zimbabwe, Harare, Zimbabwe; ^5^Perinatal HIV Research Unit, University of the Witwatersrand, Johannesburg, South Africa; ^6^Department of Psychiatry, Michigan State University, East Lansing, MI, United States; ^7^Makerere University-Johns Hopkins University Research Collaboration, MU-JHU Care Ltd., CRS, Kampala, Uganda; ^8^University of North Carolina Project–Lilongwe, Malawi CRS, Lilongwe, Malawi; ^9^Clinical Research Management, Durham, NC, United States; ^10^National Institute of Allergy and Infectious Diseases, National Institute of Health, Rockville, MD, United States; ^11^Geisel School of Medicine at Dartmouth, Hanover, NH, United States; ^12^Department of Neurology and Ophthalmology, Michigan State University, East Lansing, MI, United States; ^13^Department of Psychiatry, The University of Michigan, Ann Arbor, MI, United States

**Keywords:** pediatric HIV, antiretrovirals, neuropsychological outcomes, disease severity, predictors

## Abstract

**Background:**

The International Maternal Pediatric Adolescent AIDS Clinical Trials Network (IMPAACT) P1104s study evaluated neuropsychological outcomes over 96 weeks in children living with HIV (CLHIV) aged 5–11 years at 6 Sub-Saharan African sites to explore associations between HIV-illness related biomarkers and neuropsychological outcomes.

**Methods:**

Children living with HIV had participated in IMPAACT P1060, which compared efficacy of nevirapine versus lopinavir/ritonavir in children initiating ART at <3 years of age. At age 5–11, neuropsychological evaluations of KABC cognitive ability, TOVA attention-impulsivity and BOT-2 motor domains were assessed and repeated after 48 and 96 weeks. Clinical, antiretroviral therapy (ART) and laboratory (immunological and virological) parameters were used to predict neuropsychological outcomes using linear mixed-effects multivariable regression models, controlling for child and caregiver characteristics.

**Results:**

246 CLHIV (45% male, mean age at initial neuropsychological evaluation 7.1 yrs [SD 1.2]) began ART at a median age 14.9 months (IQR 8.2, 25.2). Nadir CD4 percentage was 14.7% (IQR 11.0, 19.5); the median peak viral load (VL) was 750 000 copies/ml (IQR 366 000, 750 000) and 63% had ≥WHO stage 3 clinical disease; 164 (67%) were on lopinavir/ritonavir, 71 (29%) were on nevirapine and 7 (3%) were on efavirenz. Other antiretrovirals were similar. Nevirapine at P1104s study start or later was associated with poorer neuropsychological scores across all domains except Global Executive Composite, even when controlling for nadir CD4 percent and time-varying HIV VL. Other predictors of poorer scores in KABC domains included low birth weight, WHO stage 4 disease and serious illness history and elevated VL was associated with worse BOT-2 scores.

**Conclusion:**

Children receiving nevirapine had poorer neuropsychological scores than those on lopinavir/ritonavir. Antiretroviral choice might adversely impact neuropsychological performance. In addition, low birth weight and markers of severe HIV disease: advanced WHO clinical HIV disease, history of serious illness and an elevated VL, were associated with lower neuropsychological scores.

## Introduction

Across 23 endemic countries, 1.5 million children still live with HIV (1). Despite global progress in early diagnosis, more access to better ART, children and adolescents living with HIV (CLHIV) still lag behind adults. Many CLHIV were born in an era when guidelines were based on cohort data and expert opinion that supported delaying ART initiation until either severely symptomatic or with CD4 + T cell depletion. Comorbidities such as tuberculosis, pneumonia and sepsis, growth failure or malnutrition, malignancy, and anemia were common in the early years of the epidemic (2–4). Almost 60 percent of CLHIV not on ART died within the first 2 years of life (5). The impact of these treatment delays and disease severity are still experienced by CLHIV as they enter school, tertiary education and the workplace in later life.

Early ART initiation has a substantial positive impact on neurocognitive function (6, 7). Numerous studies report worse neuropsychological outcomes in CLHIV than HIV-uninfected children and adolescents, specifically regarding executive functioning (8–15). Some studies report an association with clinical disease and neuropsychological outcomes, for example Ruisenor-Escudero et al., from Uganda, report worse behavioral outcomes in CLHIV with elevated viral load and CD8 + cell activation (16). Neuroimaging studies report brain volume loss and structural abnormalities correlating with neurocognitive fallout in CLHIV(14, 15). Nalwanga et al., from Uganda, reported no difference in neurocognitive results between children receiving a protease inhibitor (PI)-based regimen compared to a non-PI based regimen, however children receiving PI-based ART had better motor skills (17). However, overall data reporting associations between HIV-disease characteristics and neuropsychological outcomes are scarce. In this study we evaluated clinical, immunological, treatment related and virological factors associated with neuropsychological scores in CLHIV in IMPAACT P1104s (18–20). We hypothesized that more advanced clinical disease (advanced WHO clinical score, low anthropometrical scores, episodes of severe illness) and evidence of immune compromise with low CD4+ count, poor virological control of HIV disease with elevated HIV viral load and other laboratory abnormalities such as anemia would be associated with worse neuropsychological outcomes.

## Materials and methods

**FIGURE 1:**
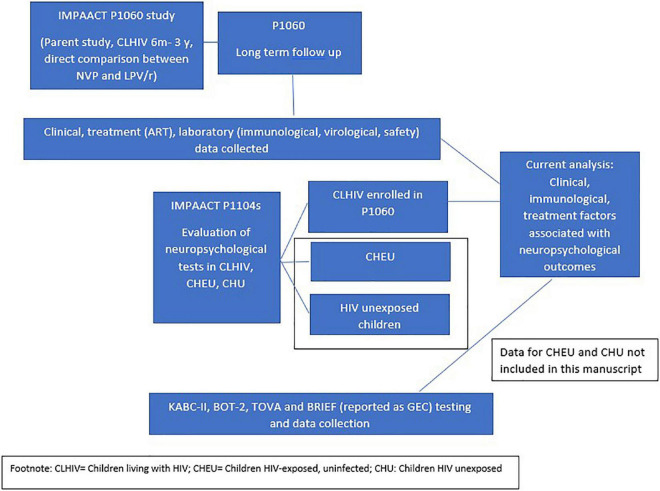
Schematically presents the P1060 study and the P1104s study, demonstrating briefly what was studied in each and how data from each study was combined for analysis in the current manuscript.

This analysis included children from two related clinical studies (Figure 1): The P1060 study (open to accrual from November 2006) and the P1104s study (opened to accrual from October 2013). P1060 enrolled CLHIV from South Africa, Zimbabwe, Malawi, Uganda, Tanzania, and Zambia who initiated ART between 6 and ≤36 months of age to compare the efficacy of nevirapine (NVP), a non-nucleoside reverse transcriptor inhibitor (NNRTI) and lopinavir/ritonavir (LPV/r), a PI. All children also received lamivudine and zidovudine on the study (19, 20). The study included children either exposed (cohort 1), or not exposed (cohort 2) to single dose NVP for vertical transmission prevention (VTP) (19, 20).

In April 2009, P1060 cohort 1 closed due to superiority of LPV/r over NVP. Cohort 2 was unblinded and closed in October 2010, again following superiority of LPV/r over NVP. The findings from P1060 have previously been reported and detailed findings are not reported here (19, 20). These outcomes were shared with parents and caregivers who were offered to switch children from NVP-based to LPV/r-based ART. Children were followed up in a longitudinal cohort on the P1060 study, receiving ART and all required HIV-related care at research site through the study.

P1104s was conducted between October 2013 and December 2016 in South Africa, Zimbabwe, Malawi and Uganda. This study compared neuropsychological outcomes in children aged 5–≤11 years from the P1060 study compared to age-matched HIV-exposed uninfected children (HEU) and children not exposed to HIV, recruited from similar neighborhoods as the CLHIV enrolled in P1060. These results have previously been reported (9, 10). Ninety-six percent of eligible P1060 participants enrolled in P1104s.

Neuropsychological evaluations were conducted at 0, 48, and 96 weeks. These evaluations included the Kaufman Assessment Battery for Children, 2nd edition (KABC-II; mental processing index or MPI score and Non-verbal Index or NVI) assessing cognitive ability, The Test of Variables of Attention (TOVA; D prime score) for measuring attention-impulsivity, the Bruininks–Oseretsky Test, 2nd edition (BOT-2) testing motor proficiency. The Behavior Rating Inventory for Executive Function (BRIEF) was used to evaluate behavioral and cognitive problems through interviewing parents or caregivers, reported as the Global Executive Composite (GEC) score.

The KABC-II is a comprehensive performance-based assessment of cognitive ability for children 3 to 18 years of age and was administered and scored using the Luria model of neuropsychological ability (21). Van Wyhe et al. (22) have documented the use of the KABC and KABC-II in its cross-cultural assessment of HIV-associated cognitive impairment in their review of published findings from numerous studies across the sub-Sahara. They concluded that the KABC/KABC-II has been used to document consistent and significant neuropsychological deficit domains in CLHIV, despite the test having been adapted in a wide range of pediatric HIV settings across different languages and cultures in various countries in southern, eastern, and central Africa (22). In the present study (P1104S, described below), Chernoff et al. (23) provided further validation of the consistency of KABC-II pediatric HIV neuropsychological assessment findings across six different study sites in four different countries (South Africa, Malawi, Zimbabwe, Uganda) adapted to eight different languages (23). A monthly quality assurance program for the KABC-II testing practices for all active testers was implemented for this study, to ensure some standardization across sites which likely significantly enhanced the strength of Chernoff and colleagues validation findings with the KABC-II in the present study (24).

Using the Luria model for administration and scoring, the KABC-II provides standardized performance measures in the neuropsychological domains of Sequential Processing (both auditory/phonological and visual-spatial working memory), Simultaneous Processing (visual spatial analysis and problem solving), Learning (along with delayed recall if needed), and Planning/Reasoning (for children 7 years of age and older). In the scoring and interpretation of KABC-II performance, these four domains (Sequential Processing, Simultaneous Processing, Learning, Planning) are combined into a composite score of overall cognitive ability, called the Mental Processing Index (MPI). That was our primary outcome in the present study, in that it is intended to be the one measure that represents overall cognitive ability performance on the KABC-II assessment battery from a neuropsychological perspective. A second composite performance measure is also available with the KABC-II call the Non-verbal Index (NVI), which is comprised of various subtests across the four domains that do not depend on spoken language or an understanding of English to complete. In addition to the MPI score, we have also sometimes used NVI an an important outcome in our P1104S and other study assessments (25, 26), when evaluating the neuropsychological impact of pediatric HIV disease over time (9, 23, 27–29). However, when selecting a single KABC-II composite performance measure *a priori* in relationship to various biomarkers and clinical predictors of pediatric HIV infection progression and severity, the measure of choice is the MPI (30).

In terms of a comprehensive assessment of neuropsychological outcomes for pediatric HIV, two important domains that are not directly assessed by the KABC-II are deficits in attention and enhanced impulsivity (attention deficit and hyperactivity disorder of ADHD), and a separate measure of overall motor proficiency. Because of the importance of these domains in assessment clinical predictors for HIV disease progress and severity in children (31, 32), we included measures of attention/impulsivity as well as motor development and performance along with the KABC-II cognitive ability test, for a complete performance-based neuropsychological assessment of our HIV-positive children in P1104s.

In the present analyses we included the Tests of Variables of Attention [TOVA version 8.0; (33)] to assess vigilance attention and impulsivity, and the short version of the Bruininks-Oseretsky Test of Motor Proficiency, 2nd edition (BOT-2). For the TOVA, our preferred single best measure of performance is the signal detection measure of D prime for the TOVA response signal. This measure combines correct responses to signal and correct non-responses to non-signal presentations, in proportion to the errors of responding to non-signal (errors of commission) along with withholding responses to signal (errors of omission).

The short version of the BOT-2 provides only one standardized measure of overall motor proficiency which includes 14 items; with one or two items pertaining to each of the following motor domains: fine motor precision, fine motor integration, manual dexterity, bilateral coordination, balance, running speed and agility, upper-limb coordination, and strength. The TOVA and BOT-2 findings for our P1104s study groups (CLHIV, HEU, HU) were included along with the KABC-II in the validation analyses by Chernoff et al. (23) and the TOVA D prime and BOT-2 total motor proficiency measure have also be reported along with the KABC-II MPI in our prior P1104s publication (9, 10, 23), and in inflammatory biomarker predictors of the P1104s CLHIV (30), as well as in other Ugandan-based neuropsychological studies of pediatric HIV (28, 29).

For all of these African-based studies in CLHIV cited above, the one non-performance-based neuropsychological measure also included in our pediatric HIV research in subSaharan Africa was the Behavior Rating Inventory of Executive Function (BRIEF) for school-age children (age 6 years or older) completed by the mother or principal caregiver of the child on study (34). This 86-item questionnaire administered to the caregiver evaluates the child’s daily behavioral and metacognitive activities at home and school, indicative of various domains of executive functioning. We used the BRIEF standardized Global Executive Composite (GEC) score in the present analyses because it is the best compositite measure of overall executive function for our P1104S study children as evaluated by the primary caregiver (35).

Clinical (anthropometry, clinical diagnoses), antiretroviral (ARV) (all previous and current ART regimens) and laboratory data (tests required for clinical management, monitoring on ART such as complete blood count, liver and renal function tests, immunological tests including CD4 + absolute counts and percentages and viral load) were collected prospectively from P1060 and this data was included for the current analysis. Clinical and laboratory events were graded according to DAIDS Table for Grading the Severity of Adult and Pediatric AEs, V1.0, 12/2004 (36). We combined these data from P1060 with clinical history, neuropsychological and caregiver data from P1104s to explore associations with clinical, treatment, laboratory data and neuropsychological outcomes. At P1104s study entry, a “serious illness history” case report form was completed. These events were tallied separately from those collected for P1060.

Linear mixed models with restricted maximum likelihood estimation (REML) and robust fixed effect error estimates were used to explore whether neuropsychological test scores were associated across time with growth, clinical history, HIV disease severity and treatment markers (VL and CD4+ percentage and count) as well as ARV regimen, and to estimate these associations. To account for participant-level variability, an intercept-only random effect term was included. Each regression analysis included a separate potential predictor. In our analysis, GEC scores were transformed so that a higher score indicated better function. To explore whether nadir CD4% or time-varying viral load may explain some of the effects of the target variables (e.g., ARV treatment), models were re-fit to include either a covariate corresponding to current log_10_ viral load or the lowest CD4% ever reported at or prior to study entry (i.e., nadir CD4%). For these analyses, we focused on three outcomes, KABC MPI, BOT-2 and TOVA D-Prime. An additional analysis that incorporated a study week by regimen interaction effect explored whether the association between ARV regimen at P1104S study entry and each neuropsychological outcome changed across the study period.

Child and caregiver characteristics, individual research sites and study week were controlled for. Slope estimates and adjusted means with 95% confidence intervals were presented. Tests of statistical significance were two-sided, and 5% error rates were used. In results tables, significant results are highlighted with shading based on the Wald test results. All models were fit using the “PROC MIXED” procedure with the “empirical” option in SAS/STAT software v9.4.

## Results

### Baseline characteristics at P1060 and subsequent P1104s entry

Baseline characteristics of all children enrolled into P1060 and then into P1104s were similar (Table 1). Of 246 CLHIV in P1104s, 45% were male, median age at entry to P1060 of 1.2 yrs (IQR 0.7, 2.1) with median age at ART initiation of 14.9 months (IQR 8.2, 25.2), the nadir CD4+ percentage was 14.7% (11.0, 19.5); the median peak VL was 750 000 copies/ml (IQR 366 000, 750 000) and 63% had ≥ WHO clinical stage 3 disease.

**TABLE 1:** Child characteristics at entry to P1060 and P1104S.

Characteristic		P1060 Entry * (*N* = 452)	P1060 Entry ** (P1104S) (*N* = 246)	P1104S Entry*** (*N* = 246)
Age (years)	Mean (s.d.)	1.4 (0.8)	1.4 (0.8)	7.1 (1.2)
	Median (Q1, Q3)	1.2 (0.7, 2.1)	1.2 (0.7, 2.1)	7.0 (6.3, 7.8)
Sex	Male	216 (48%)	111 (45%)	111 (45%)
	Female	236 (52%)	135 (55%)	135 (55%)
Race	Black African	431 (96%)	242 (98%)	242 (98%)
	Colored/White/Other	20 (4%)	4 (2%)	4 (2%)
Age started ARVs, months	Median (Q1, Q3)	14.6 (8.3, 24.9)	14.9 (8.2, 25.2)	15.0 (8.2, 25.2)
Years on entry regimen (prior)	Median (Q1, Q3)			4.6 (2.8, 5.8)
WHO disease stage	Clinical stage I	78 (17%)	28 (12%)	38 (15%)
	Clinical stage II	97 (22%)	63 (26%)	58 (24%)
	Clinical stage III	231 (52%)	136 (56%)	137 (56%)
	Clinical stage IV	41 (9%)	16 (7%)	13 (5%)
CD4% nadir	Median (Q1, Q3)	14.8 (11.0, 19.0)	14.7 (11.0, 19.5)	14 (11, 19)
CD4% nadir	0–<15%	226 (50%)	125 (51%)	131 (53%)
	15–<25%	176 (39%)	93 (38%)	92 (37%)
	=25%	49 (11%)	28 (11%)	23 (9%)
Peak log10 (RNA copies)	Median (Q1, Q3)	5.88 (5.59, 5.91)	5.88 (5.56, 5.88)	5.88 (5.59, 5.88)
CD4 count (cells/mm^3^)	Median (Q1, Q3)	970 (604, 1,414)	943.5 (624.0, 1,387.0)	1,211.5 (960.0, 1,538.0)
CD4%	Median (Q1, Q3)	16.3 (12.0, 21.6)	16.5 (12.0, 22.0)	38.6 (34.0, 43.0)
WHO weight z-score	Median (Q1, Q3)	−1.6 (−2.7, −0.6)	−1.7 (−2.8, −0.9)	−0.7 (−1.3, −0.2)
WHO height z−score	Median (Q1, Q3)	−2.3 (−3.3, −1.2)	−2.4 (−3.2, −1.4)	−1.1 (−1.7, −0.5)
WHO BMI z−score	Median (Q1, Q3)	−0.2 (−1.2, 0.7)	−0.3 (−1.4, 0.6)	−0.2 (−0.8, 0.4)
P1060 Entry ARV regimen type	NRTI + PI (LPV/r)	221 (49%)	119 (48%)	164 (67%)
	NRTI + NNRTI (EFV)	0 (0%)	0 (0%)	7 (3%)
	NRTI + NNRTI (NVP)	227 (50%)	126 (51%)	71 (29%)
	Non-HAART	1 (0%)	1 (0%)	4 (2%)
	None	3 (1%)	0 (0%)	0 (0%)

*Includes all children enrolled into P1060 at baseline/enrollments.

**Includes the baseline characteristics of all children enrolled in P1060 at baseline, who went on to enroll in 1104s.

***Includes the baseline characteristics of all children at enrollment to 1104s.

Missing values: P1060 Entry: Age, N = 3; WHO disease stage, N = 5, CD4% nadir, N = 1, CD4 count, N = 1, CD4%, N = 1, WHO weight, height, BMI, N = 1 each; P1060 Entry (P1104S); WHO disease stage, N = 3; WHO weight, height, BMI, N = 1 each.

At P1104s entry, children had been on ART for a median of 4.6 years and had higher weight-for-age and height-for-age z-scores than at entry to P1060. ART regimens at P1104s entry were as follows; 164 (67%) were receiving LPV/r, 71 (29%) were on NVP and 7 (3%) children were on EFV (NNRTI)-based ART. More children were receiving LPV/r-based regimens at P1104s baseline than on P1060 entry.

### Progress on the P1104s study

During the P1104s study, clinical, immunological, and virological characteristics remained stable across the week 48 and 96 time points. There were few regimen changes in the year preceding the study and during the P1104s study (Table 2).

**TABLE 2:** Clinical, immunological and virologic progress on the P1104S study.

		Study week		
		
Characteristic		0 (*N* = 246)	48 (*N* = 245)	96 (*N* = 242)
WHO height for age for age z-score	Mean (s.d.)	−1.04 (0.97)	−0.99 (0.99)	−1.03 (0.93)*
WHO BMI z-score	Mean (s.d.)	−0.18 (0.85)	−0.38 (0.88)	−0.48 (0.93)*
CD4+ count	Median (Q1, Q3)	1,212 (960, 1,538)	1,160 (869, 1,464)	1,105 (856, 1,496)
CD4%	Median (Q1, Q3)	39 (34, 43)	38 (33, 42)	39 (34, 43)
Log10 HIV RNA copies	Median (Q1, Q3)	1.60 (1.60, 1.75)	1.60 (1.60, 1.60)	1.60 (1.60, 1.60)
Viremic status within 9 months of visit	Aviremic	217 (88%)	223 (91%)	219 (90%)
	Viremic	29 (12%)	22 (9%)	23 (10%)
Cumulative regimen class switches	No	172 (70%)	166 (68%)	162 (67%)
	Yes	74 (30%)	79 (32%)	80 (33%)
Regimen switch in year prior	No	244 (99%)	238 (97%)	234 (97%)
	Yes	2 (1%)	7 (3%)	8 (3%)
Regimen	NRTI + PI (LPV/r)	164 (67%)	162 (66%)	164 (68%)
	NRTI + NNRTI (EFV)	7 (3%)	10 (4%)	9 (4%)
	NRTI + NNRTI (NVP)	71 (29%)	66 (27%)	64 (26%)
	Non-HAART	4 (2%)	6 (2%)	5 (2%)
	None	0 (0%)	1 (0%)	(0%)

*Denotes 1 missing value.

NRTI + PI (LPV/r) = zidovudine + lamivudine + lopinavir/ritonavir; NRTI + NNRTI (EFV) = zidovudine + lamivudine + efavirenz; NRTI + NNRTI (NVP) = zidovudine + lamivudine + nevirapine; Non-HAART included a lopinavir/ritonavir-containing regimen, but did not meet the definition for a triple ART regimen.

### Factors associated with neuropsychological scores on P1104s

Clinical risk factors (Table 3) for lower neuropsychological scores included low birth weight (LBW) for the KABC NVI score [−5.71 (−10.46, −0.96)], premature birth for the BOT-2 score [−2.67 (−4.94, −0.4)], invasive bacterial disease for GEC score [−4.96 (−9.27, −0.64)] and ADHD [−1.51 (−2.85, −0.17)], low WHO BMI z-score for D-prime [−1.80 (−3.28, −0.32)] and history of at least one serious illness event [−2.43 (−4.80, −0.06)] for GEC score. Low height for age z-score was associated with higher KABC MPI and BOT-2 scores, pulmonary disease was associated with higher KABC NVI and MPI scores, history of malnutrition with higher BOT-2 scores.

**TABLE 3:** Clinical factors associated with neuropsychological scores in 1104S.

Test variable	KABC NVI	KABC MPI	BOT-2	BRIEF GEC	TOVA ADHD	TOVA D-Prime
WHO Height for age Z score (study entry)	1.04 (−0.35, 2.43)	1.33 (0.17, 2.48)	1.30 (0.60, 1.99)	0.26 (−0.71, 1.22)	−0.08 (−0.38, 0.22)	0.31 (−1.20, 1.81)
WHO Weight for age Z score (study entry)	1.02 (−0.43, 2.48)	0.98 (−0.25, 2.21)	0.49 (−0.38, 1.35)	0.11 (−1.11, 1.34)	−0.45 (−0.76, −0.14)	−0.86 (−2.41, 0.69)
WHO BMI Z score (study entry)	0.13 (−1.32, 1.58)	−0.18 (−1.61, 1.25)	−0.84 (−1.69, 0.00)	−0.26 (−1.49, 0.96)	−0.55 (−0.88, −0.21)	−1.80 (−3.28, −0.32)
Thrombocytopenia (prior to entry)	−2.80 (−6.84, 1.23)	−3.01 (−8.18, 2.16)	5.04 (0.34, 9.75)	−0.73 (−6.54, 5.07)	−0.47 (−1.64, 0.69)	−3.28 (−6.98, 0.43)
Invasive bacterial disease (prior to entry)	0.93 (−4.29, 6.16)	0.71 (−3.41, 4.84)	−2.26 (−5.26, 0.74)	−4.96 (−9.27, −0.64)	−1.51 (−2.85, −0.17)	−3.47 (−7.46, 0.51)
Pulmonary disease (Prior to entry)*	3.74 (0.37,7.11)	3.52 (0.52,6.53)	−0.04 (−2.15,2.08)	−1.23 (−4.47,2.00)	0.23 (−0.60,1.06)	2.22 (−1.41, 5.86)
Any serious illness history**	0.19 (−2.64,3.02)	0.12 (−2.54,2.79)	−0.62 (−2.25,1.00)	−2.43 (−4.80,−0.06)	−0.38 (−1.06,0.30)	−2.38 (−5.36,0.60)
Malnutrition history	5.66 (−0.31,11.63)	3.39 (−2.63,9.42)	3.54 (0.27,6.81)	−0.01 (−4.41,4.39)	1.12 (−0.04,2.29)	1.13 (−4.85,7.12)
Premature Birth	−2.33 (−6.30,1.64)	−1.23 (−4.63,2.16)	−2.67 (−4.94,−0.40)	−0.75 (−4.38,2.87)	−0.56 (−1.75,0.62)	−3.22 (−7.95,1.50)
Low birth weight	−5.71 (−10.46,−0.96)	−2.99 (−7.32,1.35)	−1.42 (−4.22,1.39)	0.53 (−4.75,5.81)	−0.62 (−2.18,0.94)	−1.03 (−7.26,5.20)
**Serious illness history (number of types of events)**						
1 event	−0.07 (−3.27,3.13)	−0.21 (−3.20,2.78)	−0.83 (−2.59,0.93)	−3.42 (−5.93,−0.91)	−0.29 (−1.01,0.43)	−2.91 (−6.27,0.45)
2 or more events	1.06 (−3.06,5.18)	1.22 (−2.69,5.13)	0.04 (−2.61,2.69)	0.79 (−3.05,4.63)	−0.68 (−1.95,0.58)	−0.68 (−5.08,3.73)

This table represents regression slope estimates across domains, adjusted means with 95% confidence intervals.

Shading indicates F-test results p < 0.05.

Analysis of BRIEF GEC is on transformed value (100-original GEC). Results adjusted for control variables: week, site, sex at birth, age at entry, school status, whether caregiver is biological mother, residential zone, whether in caregiver’s care for less than 5 years, socioeconomic index, Caregiver HSCL depression score, and child MICS disability and development scores at entry.

Neuropsychological tests: Behavior Rating Inventory of Executive Function (BRIEF – parent rating); Global Executive Composite (GEC); Bruininks-Oseretsky Test (2nd edition) (BOT-2) test of motor proficiency; Tests of Variables of Attention (TOVA visual) ADHD index and signal detection D prime for correct responses to signal; Kaufman Assessment Battery for Children (KABC), 2nd edition Non-verbal Index (NVI) and Mental Processing Index (MPI).

*Pulmonary disease included pathologies such as tuberculosis or pneumonia.

**“Any serious illness” refers to severe illness recorded in the child’s lifetime for example meningitis, or malaria requiring hospitalization.

HIV disease severity (Table 4) associated with neuropsychological scores included peak VL > 100,000 /ml for lower BOT-2 scores [−3.35 (−6.35, −0.36)], WHO Clinical stage 4 disease for lower KABC NVI [−10.15 (−17.09, −3.20)] and KABC MPI scores [−7.85 (−14.30, −1.39)]. A CD4 nadir < 15% was associated with higher GEC scores [2.75 (0.60, 4.91)]. Older age at nadir CD4% was associated with higher BOT-2 [0.08 (0.03, 0.13)] and D-Prime scores [0.16 (0.06, 0.26)]. Higher log_10_ VL were associated with higher KABC-MPI scores, [1.01 (0.04, 1.98)] but few children had high VL during the study (Table 3).

**TABLE 4:** HIV disease severity and factors associated with neuropsychological scores.

Test variable	KABC NVI	KABC MPI	BOT-2	BRIEF GEC	TOVA ADHD	TOVA D-Prime
Viremic status within 9 months of study visit	−0.82 (−3.13, 1.49)	−1.51 (−3.12, 0.10)	−0.05 (−1.41, 1.32)	0.03 (−1.97, 2.03)	0.28 (−0.51, 1.07)	0.29 (−2.79, 3.37)
Nadir CD4%	0.07 (−0.13, 0.27)	0.03 (−0.16, 0.21)	0.08 (−0.03, 0.19)	−0.22 (−0.39, −0.06)	0.00 (−0.05, 0.05)	0.12 (−0.12, 0.36)
Nadir CD4% < 15%	−0.74 (−3.35,1.87)	0.25 (−2.06,2.56)	−0.83 (−2.49,0.83)	2.75 (0.60,4.91)	0.30 (−0.37,0.98)	−0.88 (−3.72,1.96)
Peak VL > 100K	−1.03 (−6.88,4.83)	2.01 (−2.92,6.94)	−3.35 (−6.35,−0.36)	1.21 (−2.56,4.98)	−0.16 (−1.31,0.99)	−0.09 (−6.36,6.18)
Age at nadir CD4%	0.10 (−0.00,0.21)	0.06 (−0.06,0.18)	0.08 (0.03,0.13)	−0.04 (−0.13,0.04)	0.01 (−0.02,0.03)	0.16 (0.06,0.26)
Age at peak VL	−0.00 (−0.13,0.12)	−0.01 (−0.14,0.11)	0.02 (−0.04,0.07)	−0.00 (−0.11,0.10)	0.00 (−0.03,0.03)	−0.03 (−0.17,0.12)
Log 10 VL (time-varying)	0.35 (−0.89,1.59)	1.01 (0.04,1.98)	−0.24 (−1.04,0.56)	0.24 (−0.88,1.37)	0.27 (−0.11,0.65)	0.33 (−0.99,1.64)
**WHO HIV stage**						
Clinical stage II vs. I	−0.64 (−5.82,4.55)	−0.49 (−5.47,4.50)	−0.17 (−3.29,2.95)	4.04 (−0.26,8.34)	−0.27 (−1.59,1.04)	−1.45 (−8.55,5.64)
Clinical stage III vs. I	−0.72 (−5.35,3.92)	−0.53 (−5.23,4.18)	−0.07 (−3.05,2.91)	4.43 (0.23,8.62)	0.39 (−0.87,1.64)	0.74 (−6.51,8.00)
Clinical stage IV vs. I	−10.15 (−17.09,−3.20)	−7.85 (−14.30,−1.39)	−2.25 (−6.46,1.96)	2.07 (−3.17,7.31)	−1.01 (−3.01,1.00)	−5.58 (−14.50,3.33)

Shading indicates F-test results p < 0.05.

This table represents regression slope estimates across domains, adjusted means with 95% confidence intervals.

Analysis of BRIEF GEC is on transformed value (100-original GEC). Results adjusted for control variables: week, site, sex at birth, age at entry, school status, whether caregiver is biological mother, residential zone, whether in caregiver’s care for less than 5 years, socioeconomic index, Caregiver HSCL depression score, and child MICS disability and development scores at entry.

Neuropsychological tests: Behavior Rating Inventory of Executive Function (BRIEF – parent rating); Global Executive Composite (GEC); Bruininks-Oseretsky Test (2nd edition) (BOT-2) test of motor proficiency; Tests of Variables of Attention (TOVA visual) ADHD index and signal detection D prime for correct responses to signal; Kaufman Assessment Battery for Children (KABC), 2nd edition Non-verbal Index (NVI), and Mental Processing Index (MPI).

**FIGURE 2:**
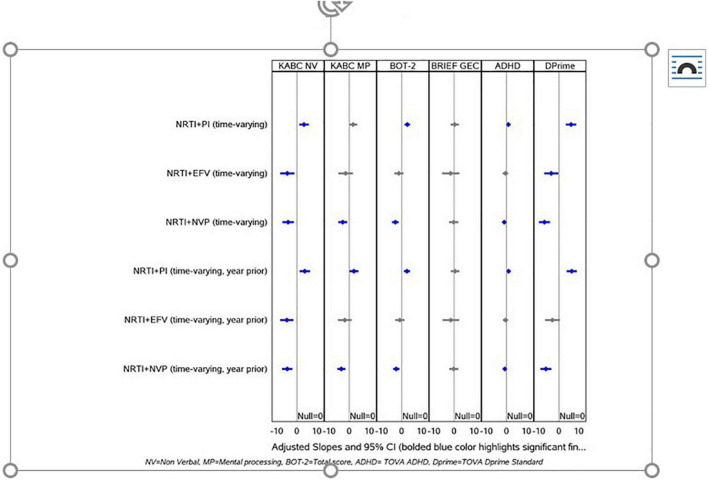
Shows the results for the current and prior year ARV exposures across all study visits. Participants whose current regimen was NNRTI and NVP-based had, on average, lower scores for KABC NVI, KABC MPI, BOT-2, TOVA ADHD, and D-Prime compared to other regimens. Conversely, those on NRTI + PI regimens had higher scores (significant or trending significant) for KABC NVI, MPI, BOT-2, TOVA ADHD, and D-Prime. Participants with EFV-based NNRTI regimens had lower scores, similar or slightly lower in magnitude to those with NVP-based regimens, with significant findings for KABC NVI and TOVA D-Prime however the number of observations for those participants on EFV-based regimens were very low. When we analyzed the treatment exposures during the year prior to the study visits, these patterns were maintained.

For all neuropsychological tests except the GEC, LPV/r was associated with higher neuropsychological scores than NVP (Figure 2 and Table 5) whether looking at current exposure at each visit or exposure in the year preceding each visit. These findings were consistent in children who had initiated PI-based therapy, and those who switched from NNRTI-based therapy and they remained consistent when controlling for nadir CD4+ percent and time-varying VL.

**TABLE 5:** ARV exposure associated with neuropsychological scores.

Test variable	ART	KABC NVI	KABC MPI	BOT-2	GEC	ADHD	D-Prime
NVP exposure (P1060)	NVP Exposed	−1.40 (−4.49,1.69)	−0.61 (−3.58,2.36)	0.94 (−0.74,2.61)	−0.53 (−3.04,1.99)	0.27 (−0.45,1.00)	0.38 (−3.10,3.86)
Time on ARV regimen at entry (years)	continuous	−0.02 (−0.67,0.62)	−0.12 (−0.72,0.48)	0.01 (−0.39,0.42)	−0.13 (−0.73,0.47)	−0.04 (−0.22,0.13)	−0.26 (−0.95,0.44)
Regimen switch, P1060 entry - > P1104S entry	PI to NNRTI	−4.18 (−10.11,1.75)	−2.67 (−8.60,3.26)	−6.29 (−10.76,−1.83)	0.68 (−3.88,5.24)	−1.45 (−3.32,0.42)	−9.59 (−18.75,−0.42)
	NNRTI to PI	−1.25 (−4.41,1.92)	0.15 (−2.89,3.19)	−1.58 (−3.75,0.58)	1.24 (−1.24,3.73)	−0.33 (−1.07,0.40)	−1.40 (−4.76,1.96)
	No switch, NNRTI	−6.50 (−9.29,−3.70)	−4.62 (−7.15,−2.08)	−3.49 (−5.24,−1.73)	0.03 (−2.47,2.54)	−1.16 (−1.94,−0.38)	−7.74 (−10.71,−4.76)
	Other switch	0.97 (−3.69,5.63)	2.36 (−3.36,8.07)	1.51 (−1.58,4.60)	3.17 (−0.93,7.27)	−0.59 (−2.30,1.11)	−0.77 (−5.94,4.40)
	No switch, PI	Ref	Ref	Ref	Ref	Ref	Ref
NRTI + PI (time-varying)		3.61 (1.17,6.05)	1.90 (−0.12,3.93)	2.81 (1.40,4.22)	0.33 (−1.76,2.43)	0.92 (0.28,1.56)	6.08 (3.50,8.67)
NRTI + EFV (time-varying)		−4.83 (−8.41,−1.24)	−1.81 (−5.48,1.87)	−1.40 (−3.66,0.86)	−1.70 (−6.04,2.64)	−0.56 (−1.91,0.79)	−3.83 (−7.31,−0.35)
NRTI + NVP (time-varying)		−4.35 (−7.22,−1.49)	−3.30 (−5.53,−1.08)	−3.18 (−4.85,−1.51)	−0.20 (−2.58,2.17)	−1.10 (−1.84,−0.36)	−7.27 (−10.01,−4.54)
NRTI + PI (time-varying, year prior)		4.02 (1.44,6.59)	2.34 (0.13,4.55)	2.65 (1.13,4.18)	0.50 (−1.64,2.65)	1.04 (0.38,1.70)	6.44 (3.88,9.01)
NRTI + EFV (time-varying, year prior)		−5.05 (−8.37,−1.73)	−2.28 (−5.73,1.17)	−0.91 (−3.29,1.47)	−1.68 (−5.86,2.50)	−0.49 (−1.81,0.83)	−3.25 (−7.01,0.51)
NRTI + NVP (time-varying, year prior)		−4.82 (−7.41,−2.23)	−3.99 (−6.00,−1.98)	−2.71 (−4.33,−1.10)	−0.18 (−2.50,2.14)	−0.83 (−1.62,−0.04)	−6.54 (−9.32,−3.77)

Shading indicates F-test results p < 0.05.

This table represents regression slope estimates across domains, adjusted means with 95% confidence intervals.

Analysis of BRIEF GEC is on transformed value (100-original GEC). Results adjusted for control variables: week, site, sex at birth, age at entry, school status, whether caregiver is biological mother, residential zone, whether in caregiver’s care for less than 5 years, socioeconomic index, Caregiver HSCL depression score, and child MICS disability and development scores at entry.

ART regimens: NRTI regimens included zidovudine and lamivudine for the P1060 study. NRTI and PI included this NRTI backbone with lopinavir/ritonavir. NRTI and EFV or NVP included efavirenz and nevirapine respectively, with zidovudine and lamivudine.

**FIGURE 3:**
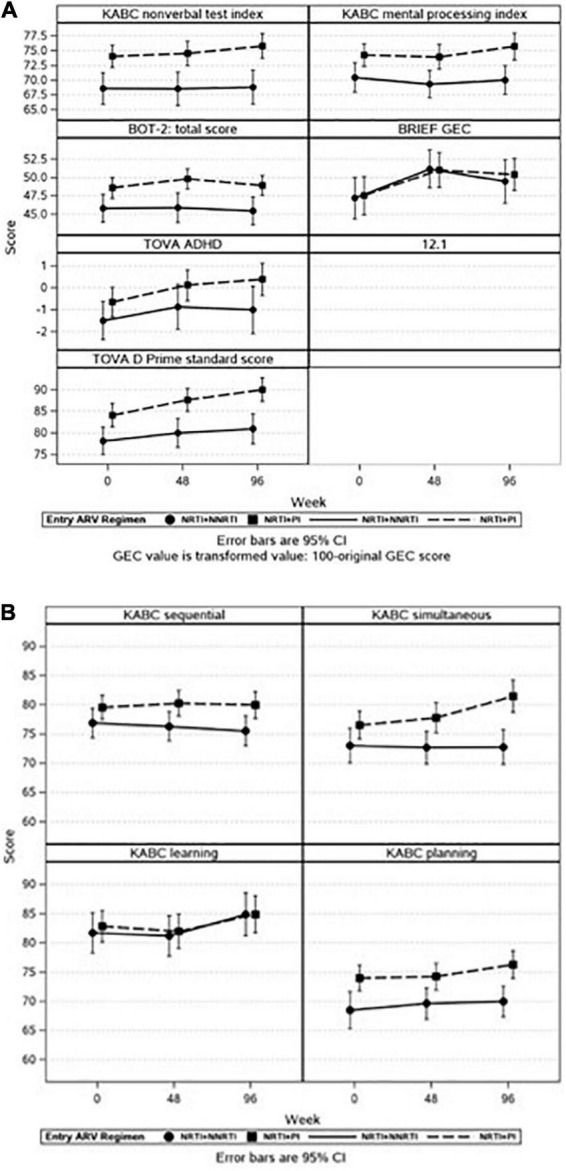
**(A,B)** It illustrates adjusted means with 95% confidence intervals (CI) for selected neuropsychological outcomes across time by entry ARV regimen. There was increased risk for lower neuropsychological scores in all domains except for the BRIEF GEC if a child was receiving NRTI + NNRTI rather than NNRTI + PI at P1104s start. Each panel represents a different outcome. **(A)** Represents the full battery of neuropsychological tests, **(B)** represents the KABC subscales.

Similar effects were observed when we explored the association between ARV regimen at the start of P1104S and neuropsychological outcomes across time. Participants on PI-based regimens had higher scores compared to NNRTI-based regimens for all tests except the GEC. Participant scores improved over time on the KABC MPI, GEC, and TOVA Attention Deficit Hyperactivity Disorder (ADHD) and D-Prime scores but similarly so for both study groups, with no statistically significant time by group interactions (Figure 3A). Participants in the PI-based regimen group had higher KABC subscale scores for sequential, simultaneous, and planning but not for learning. For both study groups, there were improvements across time for all subscales but sequential and a significant interaction effect which reflected greater improvement over time on the simultaneous domain for the PI-based regimen compared to the NNRTI-based regimen group (Figure 3B). Time on ART was not associated with neuropsychological outcomes.

## Discussion

This study provides data from a well-characterized, randomized pediatric CLHIV cohort initiating ART between 6 months and 3 years and includes clinical, HIV disease-related and ART-associated risk factors for neuropsychological test scores. Unexpectedly, NNRTI-based ART was a consistent risk factor for lower neuropsychological scores than PI-based ART across all domains except executive functioning when tested between 5 and 11 years of age. This finding was apparent at baseline, P1104s start and remained consistent at the 48-week and 96-week time-points in children who had initiated either LPV/r or NVP in P1060 and those who switched from NNRTI-based to PI-based ART prior to enrollment in P1104s. In children who switched from NVP, few switches occurred the year prior to P1104s start and almost no children switched during P1104s, therefore most were stable on a LPV/r regimen at P1104s start.

As only a small number of children received efavirenz in this study, comparisons for EFV are not possible. However these findings support a class effect for NNRTI-based ART. Nalwanga et al., from Uganda, compared neurocognitive scores in children receiving PI- versus non-PI (nevirapine or Efavirenz-based) ART. They found higher BOT-2 scores in those receiving PI-based ART, indicating improved motor function. Although there were no differences in the KABC or TOVA scores, this study was limited by a small sample size, less than a third of P1104s (17).

Children randomized to the NVP arm in early childhood, and then maintained on that arm until P1104s study entry at school age, do most poorly across all the global performance-based neuropsychology measures (KABC NVI and MPI, BOT-2, TOVA ADHD, and D-Prime) compared to children who remain on a PI-based regimen. Although there were very few, the next most poorly performing group at P1104s study entry are those children switched to NVP from their initial PI treatment regimen in P1060.

Most of the PI vs. NVP-based between-group differences in KABC neuropsychological scores averaged around 5 standard points across the three P1104s assessment points, with no overlap in the corresponding 95% CIs. Based on our prior published validation work with the KABC assessment in P1104s (23), this indicates a moderate to significant effect in terms of the clinical importance of these neurocognitive functional differences for the children in their school-age years. This clinical interpretation is based in part on US normative means and standard deviations (mean = 100; SD = 15) for the KABC NVI and MPI global performance measures (21).

These same conclusions are evident for the KABC performance domains of Simultaneous Processing and Planning especially across the three assessment points as the children get older in P1104s. Our past work with CLHIV in sub-Saharan Africa have related these differences to VL (29), and meaningful differences in quality of home environment and school performance in such children (37, 38).

Although we are unclear exactly why PI-based rather than NVP-based ART had more neuropsychological benefit, some considerations follow. In adults, detectable VLs have been associated with smaller hippocampal and amygdala volumes on magnetic resonance imaging (39). ARV levels in the cerebrospinal fluid (CSF) may indicate control of HIV replication in the brain and lower levels of penetration may increase risks of neurocognitive disorders (40). However, NVP has higher plasma to CSF concentration ratios than most other ARVs: 7.5–16.9 μmol/ml (plasma) compared to 1.3–10.9 μmol/ml (CSF), which suggests that intra-cerebral NVP levels should be adequate (40). Although plasma:CSF ratios for LPV are lower than for NNRTIs, CSF LPV levels are sufficient to suppress HIV viral replication (41). We did not measure intracerebral VL in our study, but since the NNRTIs have adequate blood brain penetration, it seems unlikely that elevated VL in the CSF accounted for this finding (40). It seems more likely associated with subsequent neuroinflammation or unrecognized toxicity. The neurological side effects of efavirenz are well described and are linked to poor school performance in Tanzanian school children (42), but neurotoxicity is uncommon with NVP, with hepatotoxicity and cutaneous toxicity far more commonly described, particularly in women with a CD4+ count of >250 cells/mm^3^ (43). NVP is a benzodiazepine derivative, and although cognitive effects occur in adults on benzodiazepines, the extent to which NVP may have a similar effect in children, is unclear (44).

Due to the findings of P1060 where LPV/r-based ART was superior to NVP-based ART, regardless of VTP exposure, PI-based ART has been recommended in children less than 3 years since 2013 (19, 20, 45). In P1060, virological failure occurred more often in children randomized to NVP than LPV/r (43/229 vs. 16/222: *p* = 0.0002, Fishers Exact 2-tail test) (46). However, in our study, time-varying VL was similar between CLHIV receiving NVP or LPV/r.

Aside from possible neurotoxic or CSF VL suppression when comparing ART treatment regiments in early childhood, poorer clinical outcomes in early childhood associated with the NVP treatment arm in P1060 (20), very likely caused a poorer neurodevelopmental trajectory throughout childhood. Even if children switched to a PI-based regimen with good clinical outcomes (e.g., long-term outcomes and viral suppression) on P1060, by the time a child entered P1104s at school age, the “die was already cast” (legacy effect) for poorer neurodevelopmental outcomes. The brain/behavior development of the child initially treated on NVP in early childhood, may have been at a neuropsychological disadvantage even if they responded well to their treatment. Children treated in very early childhood with an NRTI + PI regimen, with maintained viral suppression and a good clinical response to that regimen through early childhood, are on a much better neuropsychological trajectory with almost normal performance on the KABC and TOVA (compared to non-infected reference children in these settings) when tested in their school-age years. That is, if they are protected from other brain insults and injury from CNS infectious diseases (e.g., cerebral malaria; encephalitis, or meningitis) (47).

Nevirapine has been replaced by LPV/r and dolutegravir in most countries, as recommended by international and country-specific guidelines. NVP is still recommended in premature infants and neonates less than 2 weeks post-gestational age, with raltegravir as an alternative option. Infants are switched to LPV/r weeks to months later depending on in-country guidance. In some countries children may still receive NVP as part of a fixed dose combination therapy where either LPV/r or dolutegravir are unavailable. Additionally, NVP is widely used for postnatal prophylaxis in HIV-exposed infants for at least 6 weeks (48–50). Although NVP is infrequently used in older children, millions of children and adolescents with vertically acquired HIV, many of whom are now adults, have been exposed to NVP. Screening for neurocognitive problems in children, adolescents or adults living with HIV may identify those who could benefit from rehabilitation, however in countries with the highest HIV burden, capacity for screening and therapy remain limited. Research exploring the extent of neuropsychological impact of NVP and other factors, remains important in estimating the size of the problem, and proposing potential screening and treatment programmes that can be scaled up for maximum public health impact. Our findings suggest that neurocognitive evaluations should be considered for children receiving NVP, to advance the prevention, treatment and rehabilitation of those with disabilities. For example, computerized cognitive rehabilitation training has proven to be an entertaining and effective intervention to improve attention working memory and problem solving skills in HIV-affected schooschool-age children (51).

The GEC was not different between groups, despite differences shown in other tests which measure some elements of Executive Functioning (TOVA ADHD, TOVA D-prime, KABC simultaneous, sequential, and planning subscales). It is likely that the BRIEF was a not sensitive enough measure as it is reliant on parent/caregiver report, compared to the other tests which were from direct observation and measurement of abilities.

Additional factors associated with lower neuropsychological scores included those indicating more severe clinical and immunological HIV disease. WHO stage 4 disease was strongly associated with worse outcomes, as was any history of serious illness or invasive bacterial disease. Low weight for age z-score and BMI was also associated with worse outcomes. These factors are frequently associated with late HIV diagnosis in children (aged 8–19 years) (52, 53) and are also associated with higher morbidity and mortality even on ART (3). The findings re-emphasize the importance of early HIV diagnosis, early linkage to care and ART initiation and retention in care for children. Children in this study, initiated ART between 6 months and 3 years, at a median 14.9 months (IQR 8.2, 25.2), which is late by current standards. In addition, many children entered P1060 with advanced clinical and immunological disease and high VL (Table 1) (19). At initiation, most children had ≥ WHO stage 3 disease, a median CD4+ percentage of 16.3%, considered severely immunocompromised and a median peak VL of 5.88 log_10_ copies. The CHER study, where children were randomized to initiate on ART with first early diagnosis between 6 and 12 weeks or later based on clinical and immunological guidance at the time, early ART prevented neurocognitive delay, although locomotor scores were lower in CLHIV than controls in early years. All children were on LPV/r (7).

Undoubtably, the key issue in neuropsychological function preservation in children is early HIV diagnosis and treatment, ideally through birth diagnosis or within the first few weeks of life. Not only does this limit HIV-encephalopathy and neurocognitive impairment, but also all the comorbidities associated with untreated HIV infection (6, 7, 54). Brain structure and integrity are central to neurocognitive development and neuroimaging studies have shown a number of abnormalities in CALHIV that can explain these deficits. These include damage to neural microstructure, lower gray and white matter volume, with increased white matter hyperintensity (55). In addition, common findings are enlargement of ventricles, atrophy of the cortex and subcortex, basal ganglia abnormalities, calcifications, and corpus callosum damage (56). While the relationship between neuroimaging abnormalities and neurocognitive evaluation are not completely understood, studies have linked these findings to worse neuropsychological outcomes in CLHIV (56). Even in CLHIV started on ART early, for example, in the CHER study, 20/133 children who initiated ART at a median of 9 weeks developed HIV encephalopathy at a median of 31 weeks despite receiving suppressive therapy (57). The majority demonstrated improvement by 6 years of age, postulated to be due to resolution of intrathecal inflammation. However, recovery was poor in those with impaired brain growth (57). This emphasizes that even with relatively early ART, a significant proportion of CLHIV will have neurocognitive challenges. Reinforcement of early regular infant HIV testing in the first weeks and months of life, with early ART initiation is essential to prevent long-term neuropsychological deficits.

Prematurity and LBW were also associated with lower neurocognitive scores. Prematurity, in the absence of HIV, is associated with lower cognitive scores in children entering school, directly proportionate to the gestational age and maturity at birth (58). In turn, infants exposed to HIV are at higher risk of LBW and prematurity, which may be related to maternal HIV infection or to ART exposure, although the mechanism is not well understood (59, 60).

Surprisingly, thrombocytopenia, pulmonary disease, malnutrition, low height-for-age z-score were statistically protective for some of the neuropsychological scores. These factors usually result in worse outcomes even once initiated on ART (3) and are difficult to explain. A potential reason could be that children with these characteristics may have been referred earlier for HIV testing and entry into P1060. As these children were sick, caregivers may have been more motivated to give ART to their children and may have seen clinicians more frequently, with more nurturing interactions. In addition, it is possible that children had good recovery over time, resulting in normal scores. We did not find associations with low weight-for age or BMI, anemia, or low nadir CD4+ count or percentage, or time on ART, all of which we had predicted, given that these factors are associated with poor outcomes especially in young children. It is possible that there was a survival bias in that children who had these co-morbidities at baseline did not survive to receive ART.

Our study is not without statistical limitations. In this analysis, we screened many potentially predictive variables and did not adjust for multiple testing. In addition, the P1104S study was not powered for this secondary study objective. As a result, we could have observed spurious significant results. However, we considered this an exploratory analysis and looked for consistency in the findings. Except for our analysis of entry ARV regimens, we did not explore how associations between screening variables and neuropsychological outcomes may have changed over time since our aim was to understand broadly the overall associations between screening variables and neuropsychological outcomes.

HIV has shifted from being a severe, frequently fatal disease to a manageable chronic disease. More CLHIV are entering older childhood, adolescence, and adulthood. As neurocognitive scores in CLHIV tend to be lower than HEU and HU children, early recognition and supportive therapy remain important but are often limited in countries with the highest burden of HIV disease (9, 29). Improved neuropsychological health in children and adolescents living with HIV will improve the chances of higher functioning and productivity as adults, allowing meaningful contribution and improved quality of life.

## Conclusion

Use of NVP at P1104s study start or during follow-up was associated with poorer neuropsychological scores than LPV/r, even when controlling for nadir CD4+ percent and time-varying HIV VL. Other predictors of poorer scores in KABC domains included LBW, WHO stage 4 disease and serious illness history but not elevated VL on P1060 or P1104s. Our findings have important implications on neurocognitive function among CLHIV as newer ARVs become available. In addition, low birth weight and markers of severe HIV disease: advanced WHO clinical HIV disease, history of serious illness and an elevated VL, were associated with lower neuropsychological scores (4841).

## Data availability statement

The original contributions presented in the study are included in the article/supplementary material, further inquiries can be directed to the corresponding authors.

## Ethics statement

The studies involving human participants were reviewed and approved by relevant Human Research Ethics Committees (HRECs) in each country where the study was done. Written informed consent to participate in this study was provided by the participants’ legal guardian/next of kin.

## Author contributions

MC conducted the statistical analysis for this manuscript. LF, MFC, and MB developed the first draft and led the development of subsequent drafts of this manuscript. All authors assisted with conceptualizing this manuscript approved the manuscript and contributed significantly to the work.
